# Folic Acid Treatment Directly Influences the Genetic and Epigenetic Regulation along with the Associated Cellular Maintenance Processes of HT-29 and SW480 Colorectal Cancer Cell Lines

**DOI:** 10.3390/cancers14071820

**Published:** 2022-04-03

**Authors:** Sára Zsigrai, Alexandra Kalmár, Barbara K. Barták, Zsófia B. Nagy, Krisztina A. Szigeti, Gábor Valcz, William Kothalawala, Titanilla Dankó, Anna Sebestyén, Gábor Barna, Orsolya Pipek, István Csabai, Zsolt Tulassay, Péter Igaz, István Takács, Béla Molnár

**Affiliations:** 1 Department of Internal Medicine and Oncology, Semmelweis University, 1083 Budapest, Hungary; kalmar.alexandra@med.semmelweis-univ.hu (A.K.); molnar.barbara_kinga@med.semmelweis-univ.hu (B.K.B.); nagy.zsofia@med.semmelweis-univ.hu (Z.B.N.); szigeti.krisztina_andrea@med.semmelweis-univ.hu (K.A.S.); valcz.gabor@med.semmelweis-univ.hu (G.V.); kothalawala.william@phd.semmelweis.hu (W.K.); igaz.peter@med.semmelweis-univ.hu (P.I.); takacs.istvan@med.semmelweis-univ.hu (I.T.); molnar.bela1@med.semmelweis-univ.hu (B.M.); 2 Molecular Medicine Research Group, Eötvös Loránd Research Network, 1083 Budapest, Hungary; tulassay.zsolt@med.semmelweis-univ.hu; 3 1st Department of Pathology and Experimental Cancer Research, Semmelweis University, 1085 Budapest, Hungary; danko.titanilla@med.semmelweis-univ.hu (T.D.); sebestyen.anna@med.semmelweis-univ.hu (A.S.); barna.gabor@med.semmelweis-univ.hu (G.B.); 4 Department of Physics of Complex Systems, ELTE Eötvös Loránd University, 1117 Budapest, Hungary; pipeko@caesar.elte.hu (O.P.); csabai@phys-gs.elte.hu (I.C.); 5 Department of Internal Medicine and Hematology, Semmelweis University, 1088 Budapest, Hungary; 6 Department of Endocrinology, Semmelweis University, 1083 Budapest, Hungary

**Keywords:** colorectal cancer, folic acid, genomic stability, DNA methylation, gene expression

## Abstract

**Simple Summary:**

Folic acid (FA) participates in DNA synthesis and in DNA methylation; hence, it has a dual role in established neoplasms. We aimed to observe this phenomenon on FA-treated colorectal cancer cell lines (HT-29, SW480). Our results demonstrated that the maintenance processes, namely cell proliferation, cell viability, and DNA repair, were altered in HT-29 cells for short-term FA supplementation, while genetic and epigenetic regulations of SW480 cells were also affected. Despite the fact that FA is a precursor molecule in methyl donor formation, DNA methylation alterations were observed in both directions, primarily influencing the pathways of carcinogenesis. Moreover, behind the great number of differentially expressed genes, other FA-related effects than promoter methylation were suspected. All of our results point beyond the attributes related to FA so far. The different response of the two cell lines is worth considering in clinical practice to facilitate the effectiveness of therapy in the case of tumor heterogeneity.

**Abstract:**

Folic acid (FA) is a synthetic form of vitamin B9, generally used as a nutritional supplement and an adjunctive medication in cancer therapy. FA is involved in genetic and epigenetic regulation; therefore, it has a dual modulatory role in established neoplasms. We aimed to investigate the effect of short-term (72 h) FA supplementation on colorectal cancer; hence, HT-29 and SW480 cells were exposed to different FA concentrations (0, 100, 10,000 ng/mL). HT-29 cell proliferation and viability levels elevated after 100 ng/mL but decreased for 10,000 ng/mL FA. Additionally, a significant (*p* ≤ 0.05) improvement of genomic stability was detected in HT-29 cells with micronucleus scoring and comet assay. Conversely, the FA treatment did not alter these parameters in SW480 samples. RRBS results highlighted that DNA methylation changes were bidirectional in both cells, mainly affecting carcinogenesis-related pathways. Based on the microarray analysis, promoter methylation status was in accordance with FA-induced expression alterations of 27 genes. Our study demonstrates that the FA effect was highly dependent on the cell type, which can be attributed to the distinct molecular background and the different expression of proliferation- and DNA-repair-associated genes (*YWHAZ*, *HES1*, *STAT3*, *CCL2*). Moreover, new aspects of FA-regulated DNA methylation and consecutive gene expression were revealed.

## 1. Introduction

Folate, also known as vitamin B9, is mainly found in green leafy vegetables [1]. Its synthetic form, called folic acid (FA), is more stable chemically and has higher bioavailability than food folates [2]. FA is often used commercially in nutritional supplements and fortified products [3].

Folate mediates the transfer of one-carbon units for nucleotide synthesis and also for the formation of S-adenosylmethionine (SAM), the main methyl donor molecule responsible for most of the methylation reactions, including that of DNA [1]. Based on these facts, folate is involved in both genetic and epigenetic regulations, thereby being essential for normal cell growth and development [1]. Due to the dual modulatory effect, its role in carcinogenesis is still controversial [4], but it is becoming increasingly evident that the timing of folate supplementation is crucial [2]. Before neoplastic transformation, the FA treatment seems to be beneficial [4] because of its protective role against nucleotide imbalance, as well as the resulting DNA synthesis and repair defects [5]. In addition, FA supplementation in normal tissues can prevent global DNA hypomethylation, which is considered the hallmark of cancer by causing genomic instability [1,4]. On the other hand, in pre-existing neoplastic cells—where DNA replication is accelerated—folate has a conducive effect, since it serves as the precursor of nucleotides [2]; moreover, it may silence tumor suppressor genes by methylating their promoter region [1,6]. However, folate deficiency has the exact opposite impact to that mentioned before, both on healthy and on cancerous tissues [1]. Besides timing, the applied FA dose also has significance for tumor progression, as evidenced by numerous in vivo and in vitro experiments [7,8,9,10,11,12].

The colonic epithelium is considered to have one of the highest proliferation rates in the human body [13], and as such, it has a huge folate demand [14]. This tissue is greatly involved in tumor transformation caused by folate deficiency. As a result, the role of vitamin B9 in carcinogenesis has been intensively studied in colorectal cancer (CRC) [15]. Nevertheless, drugs interfering with folate metabolism, such as 5-fluorouracil (5-FU), are generally used in CRC treatment [16]. Chemotherapeutic regimens, such as FOLFIRI or FOLFOX, include a folate derivate, namely leucovorin, to increase the effect of 5-FU [17].

The main goal of our study was to observe the independent effect of FA on an already developed tumor by focusing on the processes in which FA has previously been reported to be involved. Hence, we investigated genetic and epigenetic regulation with transcriptome and methylome analyses, as well as the consequent maintenance mechanisms necessary for tumor growth and development, namely cell proliferation, cell viability, and DNA repair. To model the conditions of an FA-depleted diet and an adequate FA intake [8], we either exposed CRC cells to an FA-free environment (0 ng/mL) or treated them with 100 ng/mL FA. Additionally, we were interested in the beneficial or even adverse effects of extremely high FA doses on the cell lines; therefore, supraphysiological concentrations (10,000 ng/mL) were applied as well.

Since the majority of CRCs have arisen through the chromosomal instability (CIN) pathway [18], we used two CRC cell lines, namely HT-29 and SW480, representing this phenotype. Besides the similarity of CIN status, principal differences exist in respect of their molecular features, which made these cell lines suitable subjects for analyzing the dependency of the FA act on the genetic and epigenetic background. Among others, according to the consensus molecular subtype (CMS) and CpG island methylator phenotype (CIMP) classification systems, HT-29 is considered as a CMS3 (metabolic) and CIMP+, while SW480 is a CMS4 (mesenchymal) and CIMP- cell line [19,20,21]. Additionally, based on our previous whole-exome sequencing (WES) analysis [22], differences of driver mutations (HT-29: *BRAF*, SW480: *KRAS*), as well as altering mutation profiles of methylenetetrahydrofolate reductase (*MTHFR*), a key mediator gene involved in the folate cycle, were identified [22,23,24]. The latter can be accounted for approximately 50% and 70% reduction in the MTHFR activity in HT-29 and SW480 cells, respectively, thereby greatly influencing the bioavailability of the applied FA [25,26].

## 2. Materials and Methods

### 2.1. Cell Cultures

HT-29 (ATCC HTB-39) and SW480 (ATCC CCL-228) human colon adenocarcinoma cell lines were cultured in RPMI 1640 medium (LM-R1641, Biosera, Ringmer, UK) containing 10% fetal bovine serum (Biosera), 80 mg/2 mL gentamycin (Sandoz GmbH, Kundl, Austria), and 2 mM L-glutamine (Biosera). Cells were maintained at 37 °C in a 5% CO_2_ humidified atmosphere.

Prior to FA treatment, 1.25 × 10^5^ cells were seeded in triplicate in each well of a 6-well plate (Sarstedt, Nümbrecht, Germany) and incubated for 24 h in 2.5 mL growth media. For proliferation and viability assays, 96-well plates (Sarstedt) were used, and cells were seeded with a density of 3 × 10^3^ cells/well in 100 μL media.

**Figure 1:**
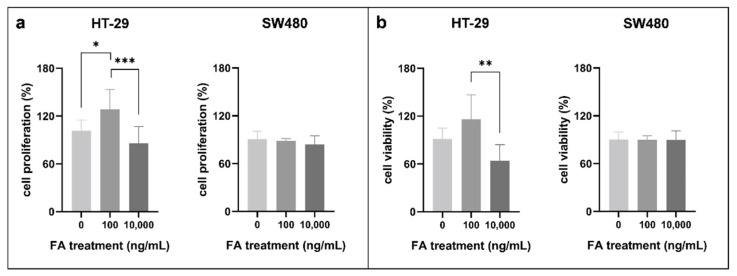
(**a**) Cell proliferation and (**b**) cell viability alterations of HT-29 and SW480 colorectal cancer cell lines following different folic acid (FA) supplies. Sulforhodamine B (SRB) was used for cell proliferation detection (* *p ≤* 0.05, *** *p ≤* 0.001), while cell viability data were obtained by alamarBlue assay (** *p ≤* 0.01). FA-depleted cells were kept in media containing 0 ng/mL FA, whereas treated cells were exposed to 100 and 10,000 ng/mL FA for 72 h. The percentages of cell proliferation and viability were given relative to samples kept in the normal growth media. FA: folic acid.

**Figure 2:**
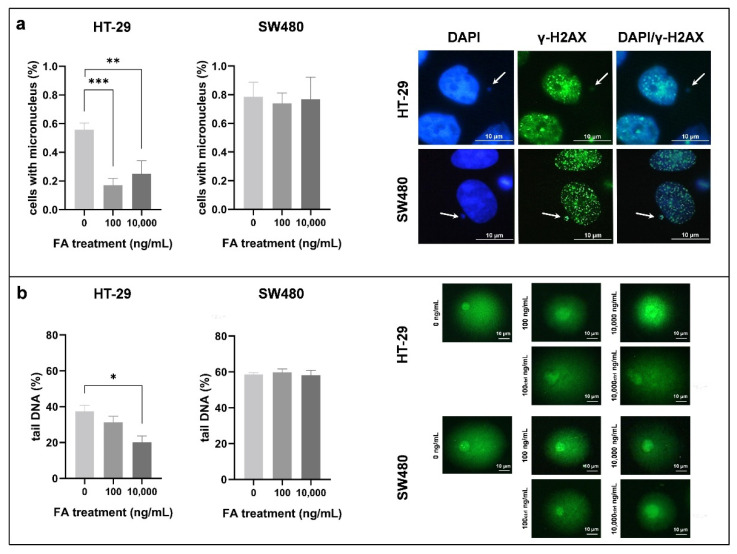
Genomic stability detection of HT-29 and SW480 cells exposed to different folic acid (FA) concentrations (0, 100, 10,000 ng/mL). (**a**) Micronucleus (MN) scoring was performed on DAPI- and anti-γ-H2AX-stained slides. Left: We obtained the results by proportioning the cells with MN with all cells counted (** *p ≤* 0.01, *** *p ≤* 0.001). Right: Representative γ-H2AX-positive micronuclei are indicated with arrows. (**b**) DNA integrity was evaluated with comet assay, additionally. Left: Graphs show the changes in genomic stability in consideration of comet tail DNA percentage (* *p ≤* 0.05). Right: Characteristic comets of both cell lines were captured following different treatments. FA: folic acid.

**Figure 3:**
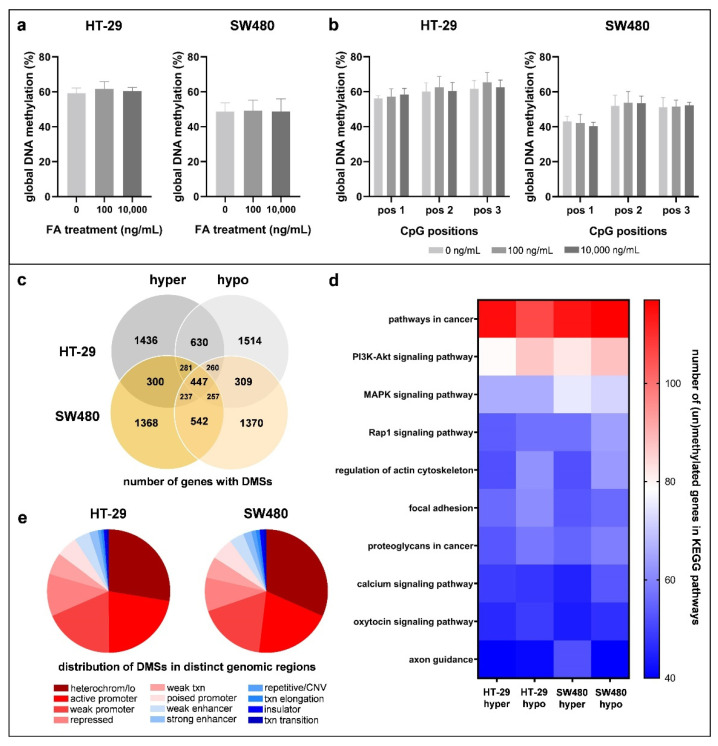
DNA methylation analysis of HT-29 and SW480 cell lines exposed to different folic acid (FA) concentrations. The methylation levels of long interspersed nuclear element 1 (LINE-1) CpG positions (pos 1, pos 2, pos 3) were (**a**) summarized and also (**b**) visualized individually to detect global DNA methylation changes. With the use of Reduced Representation Bisulfite Sequencing (RRBS) method, a genome-wide methylome profile of 10,000 ng/mL FA-treated cells was established in the comparison of cells kept in FA-free (0 ng/mL FA) media. (**c**) Firstly, the number of genes with altered methylation in the investigated CpG sites was assessed. “Hyper” and “hypo” sections indicate the number of genes with methylated and unmethylated CpG sites, respectively. The intersection of these two categories refers to the genes that possess both methylated and unmethylated CpG dinucleotides. (**d**) Heatmap shows the top 10 significantly (*p ≤* 0.05) enriched Kyoto Encyclopedia of Genes and Genomes (KEGG) pathways with the number of differentially methylated genes. (**e**) Pie charts represent the localization of differentially methylated sites (DMS) in distinct chromatin states. FA: folic acid; pos: CpG position; hyper: hypermethylation; hypo: hypomethylation; DMSs: differentially methylated sites; heterochrom/lo: heterochromatin or low signal region; txn: transcription; CNV: copy number variation; KEGG: Kyoto Encyclopedia of Genes and Genomes.

Following the 72 h long incubation period, washing with 1× phosphate-buffered saline (PBS) was performed 3 times, and the culture media were changed to FA-free RPMI 1640 (LM-R1642, Biosera). FA-depleted cells (HT-29_0_, SW480_0_) were kept in this type of media without any additional substances. Chronic supplementation can provide approximately 100 ng/mL FA concentration in human blood serum [8], and we determined the supraphysiological dose as 100 times this value; therefore, 100 ng/mL (HT-29_100_, SW480_100_) and 10,000 ng/mL (HT-29_10,000_, SW480_10,000_) FA concentrations were applied. According to the manufacturer’s instruction, FA (Sigma-Aldrich, St. Louis, MO, USA) was dissolved in 1M NaOH before it was added to the medium. After 72 h, cells were harvested with TrypLE Express (Thermo Fisher Scientific, Carlsbad, CA, USA), then they were counted to monitor cell proliferation. Trypan blue dye exclusion technique was applied to monitor cell viability. Treatment with only 1 M NaOH was also carried out in the same amount used in the case of FA supplementation to detect its individual effect on the cells. The results of control samples were subtracted from those treated with FA in order to optimize the final values. The raw data of Figures 1, 2 and 3a,b can be found in Table S1.

### 2.2. Cell Viability and Proliferation Analysis with alamarBlue and Sulforhodamine B Assays

Cell viability was measured with alamarBlue. The obtained results refer to the activity of the electron transport chain; hence, it is a so-called indicator of cell health. Furthermore, Sulforhodamine B (SRB) assay was used for cell proliferation detection, as it can estimate the protein mass of cultured cells.

AlamarBlue (Thermo Fisher Scientific) was added to the wells 4 h prior to the 72 h long incubation period. Next, the fluorescence was measured in the 570–590 nm range for cell viability detection using a fluorimeter (Fluoroskan Ascent FL Microplate Fluorometer and Luminometer, Thermo Fisher Scientific).

Following the measurement, we carried out Sulforhodamine B assay on the same plate to detect cell proliferation changes. First, cells were fixed using trichloroacetic acid for 1 h at 4 °C, then washed with tap water. Dyeing was performed with 50 μL 0.4% *m/v* sulforhodamine B solution for 15 min at room temperature (RT), and the unbound dye was removed with 1% *v/v* acetic acid. After air drying, 150 μL of 10 mM, unbuffered tris base was added to the wells, and the samples were shaken thoroughly. Finally, absorbance was measured with a microplate reader at 570 nm wavelength using Transmit software (Multiskan MCC 355, Thermo Fisher Scientific). The percentage of cell viability and cell proliferation was compared to the samples kept in the RPMI 1640 growth media. One-way ANOVA, followed by Tukey’s multiple comparisons test, was applied to assess statistical significances (*p* *≤* 0.05) using Prism 8.0.2 software (GraphPad, San Diego, CA, USA).

### 2.3. Cell Cycle Analysis with Flow Cytometry

After harvesting around 1 × 10^6^ HT-29 and SW480 cells, 1× PBS washing was performed, and cells were fixed by keeping them at −20 °C in 70% ethanol overnight. The next day, the samples were centrifuged, and the pellets were resuspended with 1× PBS. Following RNase (Thermo Fisher Scientific) treatment for 15 min, 2 μL propidium iodide (2 mg/mL) (Sigma-Aldrich) was added to the samples. FACSCalibur bench-top flow cytometer (Becton, Dickinson and Company, Franklin Lakes, NJ, USA) and CellQuest Pro software (Becton, Dickinson and Company) were used to measure the samples and analyze the results. Statistical significances (*p ≤* 0.05) were assessed by two-way ANOVA followed by Tukey’s multiple comparisons tests using Prism 8.0.2 software (GraphPad).

### 2.4. Genomic Stability Detection with Micronucleus Scoring

Micronucleus scoring was applied to detect the level of genetic damage via the analysis of chromosomes or their fragments that lagged behind during cell division. HT-29 and SW480 cells were grown on glass coverslips, placed into 6-well plates, as described before by Valcz et al. [27], and, following a 24 h long incubation period, we performed the FA treatment. Cells were fixed with 10% buffered formalin for 10 min at 4 °C, and permeabilization was carried out with 0.2% Triton-X-100 for another 10 min at RT. Anti-γ-H2AX antibody (ab26350, Abcam, Cambridge, UK) (1 h; 1:150 dilution; RT; anti-mouse) labeled with Alexa 488 (Invitrogen, Carlsbad, CA, USA) (30 min; 1:200 dilution; RT) was used for γ-H2AX detection in the micronuclei. Cell nuclei and micronuclei were stained with DAPI staining (Thermo Fisher Scientific) (5 min; 1:1000 dilution; RT). Washing with 1× PBS was performed 3 times between each step of the immunostaining. Slides were digitized in 21 Z-axial confocal layers of 0.4 μm focus steps using Pannoramic Confocal scanner (3DHISTECH Ltd., Budapest, Hungary), and an area containing at least 4000 cells per sample was analyzed. CellQuant tool of CaseViewer software (3DHISTECH) was applied to count micronucleus (MN) numbers and to obtain the percentage of cells with MN. The range within the MN number was detected was in line with the data of previous studies [28,29]. Frequently detected chromatin-containing nuclear blebs, which were not evidently separated from the nuclei, were excluded from the analysis. Statistical significances (*p* ≤ 0.05) were evaluated by one-way ANOVA followed by Tukey’s multiple comparisons test using Prism 8.0.2 software (GraphPad).

### 2.5. Genomic Stability Detection with Comet Assay

Comet assay was used for the detection of DNA strand breaks, since free DNA segments have increased migration speed during electrophoresis. At the end of the 72 h long FA treatment period, we removed the cells from the bottom of the plate with a rubber policeman, then washed the samples with Dulbecco’s PBS (Sigma-Aldrich). The Comet Assay Kit (Abcam) was applied according to the manufacturer’s protocol with a minor modification: original slides were replaced with agarose-coated Superfrost Ultra Plus slides (Thermo Fisher Scientific). Single-cell electrophoresis was performed in alkaline electrophoresis solution at 150 mA for 45 min. Vista Green Dye (Abcam) was used to stain the DNA. Comets were captured by an AxioCam camera (Carl Zeiss AG, Oberkochen, Germany) attached to a fluorescence microscope (Carl Zeiss AG). At least 50 cells/sample were analyzed using Comet Score software, and tail DNA% was defined as the ratio of tail DNA and cell DNA length. Statistical analyses were carried out using Prism 8.0.2 software (GraphPad), and significances (*p* *≤* 0.05) were assessed by one-way ANOVA test followed by Tukey’s multiple comparisons test.

### 2.6. DNA Methylation Analysis with LINE-1 Pyrosequencing

DNA isolation was performed with High Pure PCR Template Preparation Kit (Roche, Mannheim, Germany). Qubit dsDNA HS Assay Kit (Thermo Fisher Scientific) was used on Qubit 1.0 fluorometer (Thermo Fisher Scientific) for measuring the concentration of the extracted DNA; then, samples were stored at −20 °C for later analyses. Bisulfite conversion of 100 ng DNA was performed with EZ DNA Methylation-Direct Kit (Zymo Research, Orange, FL, USA). PyroMark PCR Kit (Qiagen, Hilden, Germany) was used to amplify a 146-basepair-long region of long interspersed nuclear element 1 (LINE-1) retrotransposon, and the PCR product was visualized with gel electrophoresis using 2% agarose gel. According to the instructions of PyroMark Q24 CpG LINE-1 Handbook (Qiagen), samples were prepared for pyrosequencing on a PyroMark Q24 Vacuum Workstation (Qiagen). Pyrosequencing was performed by PyroMark Q24 System (Qiagen), and LINE-1 methylation level was quantified with PyroMark Q24 Software (Qiagen). The mean methylation level of three LINE-1 CpG (cytosines followed by guanine residues) sites was interpreted as the global DNA methylation level of the given sample. Two-way ANOVA followed by Tukey’s multiple comparisons test was applied to determine statistical significances (*p* *≤* 0.05) using Prism 8.0.2 software (GraphPad).

### 2.7. DNA Methylation Analysis with Reduced Representation Bisulfite Sequencing

The isolated DNA from HT-29 and SW80 cells kept in 0 and 10,000 ng/mL FA-containing media was used for genome-wide methylation profile analysis with Premium Reduced Representation Bisulfite Sequencing (RRBS) Kit (Diagenode Diagnostics, Seraing, Belgium). After enzymatic digestion, ends preparation, adaptor ligation, and size selection, the samples were quantified with qPCR. Based on their Ct value, 2 pools were created. Following the bisulfite conversion and amplification steps, DNA was quantified with Qubit 1.0 Fluorometer using Qubit dsDNA HS Assay Kit (Thermo Fisher Scientific). Then, quality control was performed with Agilent 2100 Bioanalyzer using Agilent High Sensitivity DNA Kit (Agilent Technologies, Santa Clara, USA). The samples were prepared according to the Next Seq System Denature and Dilute Libraries Guide (Illumina, San Diego, USA), then transferred to NextSeq 500/550 High Output Sequencing kit v2 (75 cycles) (Illumina). Finally, we used NextSeq 500 instrument (Illumina) for sequencing. Sequencing data were assembled into single FASTQ files by merging the results of different sequencing lanes. As a quality assessment step, fastQC [30] was run on all resulting FASTQ files. Adapter and quality trimming was performed with Trim Galore, with default settings optimized for Bisulfite-Seq files. Prior to alignment, the hg38 version of the human reference genome was bisulfite converted in silico and indexed using the Bismark software [31]. Alignment and methylation calling were performed by the same software with default parameter values. The lists of differentially methylated sites (DMSs), along with CpG content and coverage histograms in sample pairs, were obtained with the methylKit R package [32]. The default “SLIM” method was used for multiple testing correction. The list of potential DMSs was filtered out if either the methylation difference between the two compared samples was below 15%, or the q-value of the comparison was above 0.05. Annotation of DMSs was performed using the appropriate annotation databases downloaded from the UCSC Genome Browser website [33]. The Kyoto Encyclopedia of Genes and Genomes (KEGG) pathway enrichment analysis was performed using The Database for Annotation, Visualization and Integrated Discovery (DAVID) v6.8 tool [34]. The top 10 KEGG pathways significantly (*p* *≤* 0.05) enriched for DMSs in both cell lines were determined and later illustrated with heatmaps.

### 2.8. Whole Genomic Expression Analysis with Microarray

HT-29 and SW480 cells kept in the FA-free medium or treated with 10,000 ng/mL folate were involved in this evaluation. Harvested cells were washed with 1× PBS; then, following a centrifugation step, pellets were resuspended with 350 μL RLT buffer containing β-mercaptoethanol. Total RNA was purified with RNeasy Mini Kit (Qiagen), and isolated RNA concentration was measured with Qubit 1.0 fluorometer using RNA HS Assay Kit (Thermo Fisher Scientific). Agilent 6000 Pico Assay Kit on Agilent 2100 Bioanalyzer system (Agilent Technologies) was used for determining the integrity of RNA. Samples with a higher RNA integrity number (RIN) than 8 were applied for further analysis. Using GeneChip WT PLUS Reagent Kit (Thermo Fisher Scientific), amplification, quantification, fragmentation, and terminal labeling were performed, followed by target hybridization to HTA 2.0 microarray (Human Transcriptome Array 2.0, Affymetrix, Santa Clara, CA, USA) according to the manufacturer’s instructions. Washing, staining, and scanning steps were performed as previously described by Kalmar et al. [35].

The signal space transformation–robust multi-array average (SST-RMA) algorithm was applied for background subtraction, normalization, and signal summarization. Gene expression alterations were evaluated using Transcriptome Analysis Console 4.0 (TAC 4.0, Affymetrix) software. Transcript IDs showing significant alterations (*p* *≤* 0.05) with a fold change (FC) equal or greater than 1.5 and equal or lower than −1.5 were annotated with Ensembl Genome Browser BioMart and used for further analyses. We obtained the normalized log2 values from the TAC 4.0 software, and with the use of the “prcomp” package of R, we performed principal component analysis (PCA). Protein–protein interaction (PPI) networks of differentially expressed genes were built with the StringApp of Cytoscape software. Color coding was applied for indicating the expression level of the genes (dark blue: FC ≤ −2, light blue: FC ≥ −2 and ≤ −1.5, light red: FC ≥ 1.5 and ≤ 2, dark red: FC ≥ 2). Top 10 genes showing significant (*p* *≤* 0.05) up- and downregulation, as well as genes with a promoter methylation status alteration in accordance with their expression level (*p* *≤* 0.05 and fold change ≥ |1.5|), were visualized with volcano plots using TAC 4.0. Finally, Protein Analysis Through Evolutionary Relationships 16.0 classification system (PANTHER 16.0) was applied for determining the molecular pathways in which the affected genes were involved.

## 3. Results

### 3.1. Effect of Folic Acid Treatment on Cell Proliferation, Cell Viability, and Cell Cycle

As a first step, we examined the results of FA treatment on the proliferation of two CRC cell lines using SRB assay (Figure 1a). In HT-29 cells, the highest proliferation rate was detected following 100 ng/mL FA supplementation (HT-29_100_: 128.43 ± 24.94%). Meanwhile, FA depletion (HT-29_0_: 101.25 ± 13.53%) and 10,000 ng/mL FA concentration (HT-29_10,000_: 86.06 ± 20.75%) caused significant (*p ≤* 0.05) reduction compared to this value. By contrast, remarkable alterations were not observed in SW480 cell line for different FA supplies (SW480_0_: 90.96 ± 9.72%, SW480_100_: 88.75 ± 2.69%, SW480_10,000_: 84.15 ± 10.67%).

The tendency of cell viability was analogous to the results detected during cell proliferation analyses (Figure 1b). In HT-29 cells, the values were decreased in the case of FA depletion (HT-29_0_: 91.57 ± 13.27%) and also for supraphysiological (HT-29_10,000_: 64.06 ± 20.24%) treatment compared to 100 ng/mL FA level (HT-29_100_: 115.81 ± 30.88%). However, in SW480 cells, the viability was around 90%, independent of the applied FA dose (SW480_0_: 90.22 ± 9.55%, SW480_100_: 90.05 ± 5.03%, SW480_10,000_: 89.72 ± 11.22%).

In order to examine how FA treatment affects the cell cycle, fluorescence-activated cell sorting (FACS) measurement was performed. The proportion of cells in the different cell cycle phases was specific to the given cell type, with a G0/1 phase dominance in HT-29 and an S phase dominance in SW480 cells. Interestingly, it was not affected by any treatment conditions (an average of all HT-29 samples: 62.51 ± 4.76% in the G0/G1, 17.90 ± 4.32% in the G2/M, and 19.59 ± 2.12% in the S phase; an average of all SW480 samples: 41.31 ± 1.60% in the G0/G1, 0.81 ± 0.66% in the G2/M, and 57.88 ± 1.59% in the S phase).

### 3.2. Effect of Folic Acid Treatment on Genomic Stability

We analyzed genomic stability alterations affected by FA treatment with MN scoring (Figure S1) and comet assay (Figure S2). According to immunocytochemistry (Figure 2a), 0.56 ± 0.05% of the HT-29 cells proved to have MN in the FA-depleted environment, while, as a result of FA treatment, this value significantly (*p ≤* 0.01) decreased (HT-29_100_: 0.17 ± 0.05%, HT-29_10,000_: 0.25 ± 0.09%). On the other hand, 0.79 ± 0.10% SW480 cells kept in FA-free medium had MN, and the ratio was barely affected by the FA treatment (SW480_100_: 0.74 ± 0.07%, SW480_10,000_: 0.77 ± 0.16%).

Besides DAPI staining, anti-γ-H2AX antibody was applied, and the rate of micronuclei showing γ-H2AX positivity compared to all micronuclei was determined. Approximately 79% of these particles were γ-H2AX positive, independent of the cell type or the treatment condition (HT-29: 76.68 ± 3.54%, SW480: 80.35 ± 6.46%).

In the case of comet assay, the percentage of DNA in the comet tail was the parameter used to describe DNA damage (Figure 2b). In FA-depleted HT-29 cells, tail DNA was 37.35 ± 3.45%, which was even higher in SW480 cells with a 58.79 ± 0.83% rate. Following FA supplementation, a reduction in comet tail length could be observed in HT-29 samples (HT-29_100_: 31.23 ± 3.41%, HT-29_10,000_: 20.07 ± 3.59%), while in FA-treated SW480 cells, prominent alterations were not detected (SW480_100_: 59.84 ± 1.89%, SW480_10,000_: 58.24 ± 2.56%).

### 3.3. Effect of Folic Acid Treatment on DNA Methylation

FA is involved in the DNA methylation process; thereby, the analysis of its alterations was needed. The methylation levels of three LINE-1 CpG sites were summarized (Figure 3a) and also visualized individually (Figure 3b) to assess global DNA methylation. Although HT-29 cells had an 11.59% higher DNA methylation level than SW480 samples, remarkable changes could not be detected for FA supplementation in any of the cell lines (HT-29_0_: 59.30 ± 2.90%, HT-29_100_: 61.68 ± 4.17%, HT-29_10,000_: 60.42 ± 2.12%; SW480_0_: 48.76 ± 4.93%, SW480_100_: 49.15 ± 6.13%, SW480_10,000_: 48.73 ± 7.27%).

Next, we determined DNA methylation changes with RRBS in order to compare the genome-wide methylome map of FA-depleted and 10,000 ng/mL FA-treated samples. Following FA supplementation, the number of genes with methylated (HT-29_hyper_: 1436, SW480_hyper_: 1368) and unmethylated (HT-29_hypo_: 1368, SW480_hypo_: 1370) CpG sites was similar within a cell line and between the two cell types as well (Figure 3c). KEGG enrichment analysis revealed that genes that underwent methylation changes following the FA treatment were dominantly associated with carcinogenesis in both cell lines (Figure 3d). The gene numbers in different pathways were within the range of 40–117. Based on the localization of DMSs in distinct chromosomal states, the heterochromatin or low signal regions (HT-29: 27.57%, SW480: 31.61%) along with active (HT-29: 22.29%, SW480: 20.29%) and weak (HT-29: 18.57%, SW480: 17.81%) promoter sequences were mainly represented (Figure 3e).

### 3.4. Effect of Folic Acid Treatment on Gene Expression

**Figure 4:**
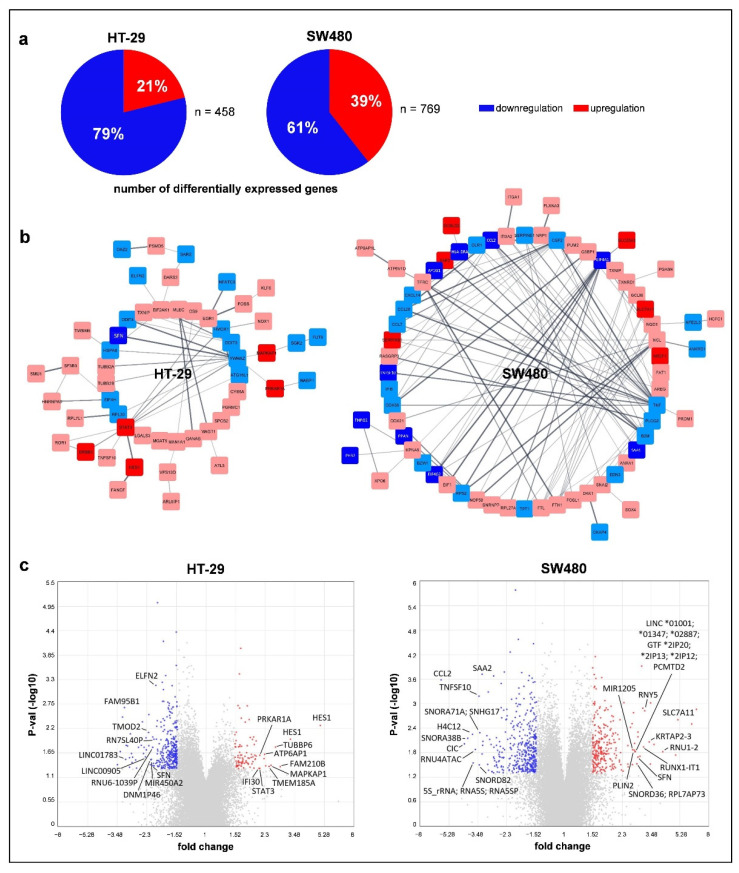
Genome-wide transcriptome alterations of HT-29 and SW480 cells following 10,000 ng/mL folic acid (FA) supplementation detected by Human Transcriptome Array 2.0 (HTA 2.0). (**a**) Pie charts represent the proportion of up- and downregulated genes. (**b**) Visual networks of protein–protein interactions were generated by the StringApp of Cytoscape software based on the list of genes with significant (*p* *≤* 0.05) expression alterations and ≥|1.5| fold change (FC). Colors refer to the expression level of protein-coding genes (dark blue: FC ≤ −2, light blue: FC ≥ −2 and ≤−1.5, light red: FC ≥ 1.5 and ≤2, dark red: FC ≥ 2). (**c**) Top 10 genes showing significant (*p* *≤* 0.05) up- and downregulation visualized with volcano plots. Gray points represent all the transcripts detected by HTA 2.0 microarray, while significantly (*p* *≤* 0.05) altering genes with FC ≥ |1.5| value were marked with red and blue. P-val: *p*-value.

Folate can influence gene expression through its interplay in epigenetic modifications. Microarray analysis of non-treated and 10,000 ng/mL FA-treated cells was performed to investigate the above-mentioned phenomenon. We focused on transcripts owning gene symbols, according to Ensemble BioMart annotation, that showed significant alterations with a fold change (FC) equal or greater than 1.5 and equal or lower than −1.5. Following FA supplementation, genes with decreased expression predominated mainly in HT-29 cells (HT-29: 78.82% down- and 21.18% upregulation, SW480: 60.60% down- and 39.40% upregulation) (Figure 4a). However, the number of altering genes (HT-29: 458, SW480: 769) (Figure 4a) and also the extent of the alterations analyzed with PCA (Figure S3) were higher in the SW480 cell line. Moreover, according to PPI networks constructed by STRING, the predicted functional associations of proteins encoded by these genes were also more complex (HT-29: 113 nodes and 82 edges, SW480: 186 nodes and 221 edges). In HT-29 cells, YWHAZ (11 edges), while in SW480, TNF (27 edges) were the hub proteins based on the number of neighbors (Figure 4b).

In HT-29 cells, two transcripts (TC03001071.hg, TC03002732.hg) detecting the *HES1* gene showed the greatest increase in their expression level after FA treatment (FC: 4.98 and 3.27), followed by *FAM210B*, *TUBBP6*, *MAPKAP1*, and *TMEM185A* (FC: 2.85, 2.65, 2.49, 2.42, respectively). On the other hand, *FAM95B1* detected by two transcripts (TC01003063.hg, TC09001384.hg) was the most predominantly downregulated gene (FC: −3.15 and −3.15) in this cell line along with *LINC01783*, *LINC00905*, *TMOD2*, and *MIR450A2* (FC: −3.09, −2.34, −2.24, −2.17, respectively). In the SW480 cell line, *SLC7A11* had the highest expression change in response to FA supplementation with a 5.12 FC, and the following four genes (*RNU1-2, KRTAP2-3*, *RNY5*, and *RUNX1-IT1*) showed higher than 3 FC (4.12, 3.40, 3.18, 3.13, respectively). The greatest gene expression decrease was observed in the *CCL2* gene (FC: −5.90), then in *CIC*, *SNORA38B*, *H4C12*, and 5s ribosomal RNA genes or pseudogenes, such as *RNA5S1*, *RNA5SP150*, *RNA5SP226*, and *RNA5SP389* (FC: −4.22, −4.01, −3.76, −3.72, respectively) (Figure 4c; Table S2). In the common set of HT-29 and SW480 cells, seven genes, namely *LOC727896*, *LOC105371030*, *RN7SL677P*, *RPPH1*, *SFN*, *TNFSF10*, and *TXNIP* were included based on our criteria (*p* *≤* 0.05 and FC ≥ |1.5|).

According to the PANTHER classification system, the “platelet-derived growth factor (PDGF) signaling” pathway was the one wherein most of the analyzed genes (*p* *≤* 0.05 and ≥ |1.5| FC) of HT-29 cells were represented (*SRGAP1*, *SRGAP2*, *SRGAP2B*, *SRGAP2C*, *STAT3*, *RAB11B*, and *FOSB*). In addition, all of these genes were overexpressed after FA treatment. Concerning SW480 cells, the “inflammation mediated by chemokine and cytokine signaling” pathway was mostly affected by FA treatment, as five genes (*CCL2*, *CCL7*, *CCL20*, *ITGA2*, and *PLCG2*) were involved. Except for *ITGA2*, all genes showed downregulation for FA supplementation.

**Figure 5:**
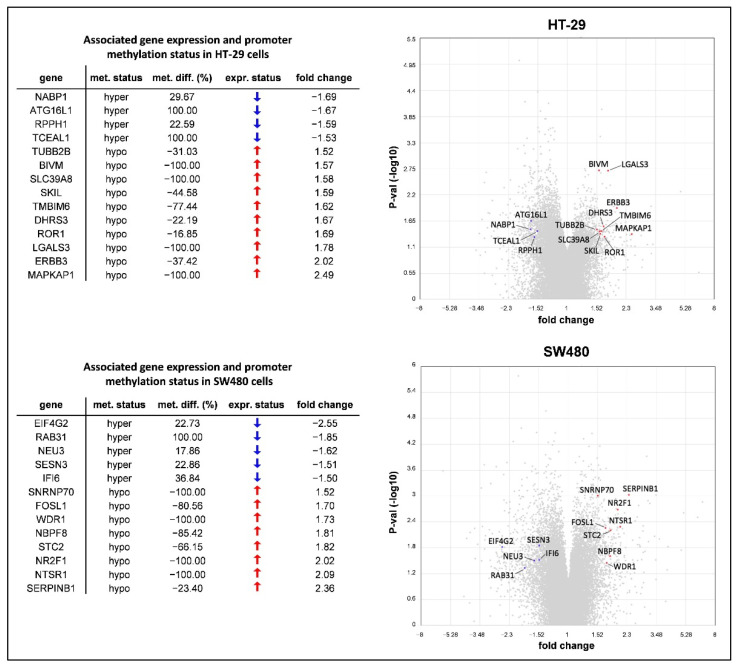
The intersection of genome-wide DNA methylation and gene expression data obtained by Reduced Representation Bisulfite Sequencing (RRBS) and Human Transcriptome Array (HTA) 2.0 analyses. Values represent the methylome and transcriptome pattern changes of 10,000 ng/mL folic acid (FA)-treated HT-29 and SW480 cells compared to non-treated samples (0 ng/mL FA). Only genes with promoter methylation status alteration in accordance with their expression level (*p* *≤* 0.05 and fold change ≥|1.5|) were listed (left) and also visualized in volcano plots (right). Gray points represent all the transcripts detected by the microarray, while blue ones highlight down- and red ones show upregulating genes from the list. met. status: DNA methylation status; met. diff.: DNA methylation difference; expr. status: gene expression status; P-val: *p*-value.

Finally, RRBS and HTA data were linked as we evaluated the expression level of genes possessing DMSs within the promoter region. Promoter methylation caused downregulation of four (*NABP1*, *ATG16L1*, *RPPH1*, and *TCEAL1*) and five (*EIF4G2*, *RAB31*, *NEU3*, *SESN3*, *IFI6*) genes in HT-29 and SW480 cells, respectively. Meanwhile, hypomethylation of the promoter region corresponded with upregulation of 10 genes (*TUBB2B*, *BIVM*, *SLC39A8*, *SKIL*, *TMBIM6*, *DHRS3*, *ROR1*, *LGALS3*, *ERBB3*, and *MAPKAP1*) in HT-29 and 8 genes (*SNRNP70*, *FOSL1*, *WDR1*, *NBPF8*, *STC2*, *NR2F1*, *NTSR1*, and *SERPINB1*) in SW480 cells (Figure 5).

## 4. Discussion

Folate deficiency has been shown to be associated with several disorders, such as megaloblastic anemia [36], cardiovascular diseases [37], obstetrical complications [38,39], neuropsychiatric conditions [40], and malignancies [41]. Its supplementation is considered to be beneficial in the prevention of all the diseases mentioned above. However, great care is required, as folate can promote the progression of established neoplasms, while the low intake has an exactly opposite effect [41,42]. FA is commonly used in CRC care as an adjunctive medication to promote the efficacy of chemotherapy medications [16,17]. However, its individual effect on FA-related cellular maintenance activities, along with the genetic and epigenetic regulation, has not been evaluated comprehensively. Therefore, we found it important to observe the short-term (72 h) impact of FA-free (0 ng/mL) and FA-supplemented (100 ng/mL and 10,000 ng/mL) environments on two CRC cell lines (HT-29 and SW480).

An adequate level of nucleotides is necessary for tumor progression, and FA can serve this need by participating in DNA synthesis [2]. This phenomenon can be in the background of the significantly (*p* *≤* 0.05) elevated HT-29 cell proliferation for 100 ng/mL FA treatment, along with an increased cell viability compared to the FA-depleted environment. These observations were in line with the results of Pellis et al. [8]. Interestingly, supraphysiological FA concentration caused lower proliferation than in FA-depleted samples. Previous studies demonstrated that similarly high FA dose inhibited the proliferation of CRC cell lines (HT-29, COLO-205, LoVo), along with endothelial and nasopharyngeal cancer cells, supposedly via the activation of folate receptor α-mediated extracellular signal-regulated kinase (ERK) pathway [7,43,44]. Conversely to HT-29 samples, cell proliferation and viability changes could not be detected in the SW480 cell line. Farias et al. likewise verified that for various FA exposures, SW480 cells had a different proliferation rate than other CRC cell lines (HCT116, LS174T) [4].

It is already known that low folate levels can lead to genomic instability through the disturbances of DNA methylation and nucleotide imbalance [45,46,47,48,49,50]. Since FA intake might improve these conditions, we examined DNA integrity using MN scoring and comet assay methods. SW480 cells inherently had greater genomic instability than the HT-29 cell line, comparing the samples kept in FA-free environment. The micronucleus number significantly decreased for FA supplementation, and comets were shortened in parallel with the applied FA dose regarding the HT-29 cell line. Therefore, the treatment facilitated genomic stability, similarly to the results of Catala et al. using colonic epithelial cells and fibroblasts [45]. On the other hand, FA treatment did not cause any remarkable changes in the genomic stability of SW480 samples.

DNA methylation—one of the most intensely studied epigenetic modifications—is crucial to normal genome regulation [51,52,53]. After FA is converted into 5-methyltetrahydrofolate (5-methyl-THF) by the MTHFR enzyme, it participates in the synthesis of SAM, the main methyl donor molecule [42]. Hence, we investigated global DNA methylation alterations with LINE-1 bisulfite sequencing in response to FA treatment. HT-29 samples had an 11.59% higher baseline DNA methylation level than SW480 cells, in agreement with their CIMP statuses [19]. However, remarkable changes were not detected in any of the samples after FA supplementation. Stempak et al. came to similar results when analyzing folate-deficient and sufficient HCT116 and Caco-2 CRC cell lines [54].

Thereafter, DNA methylation was analyzed at a higher resolution on the single-nucleotide level with RRBS method. Our results highlighted that the methylation pattern did not differ much between the two cell lines following 10,000 ng/mL FA supplementation in regard to the affected gene numbers, pathways, and genomic regions. Additionally, FA equally caused DNA hyper- and hypomethylation, as the numbers of genes possessing methylated and unmethylated DMSs were relatively equivalent. This phenomenon may explain the non-significantly altering global DNA methylation results. In the background of the discrepancy between the facts that FA is a precursor in methyl donor formation, but it also modulates DNA methylation in both directions, we suspect the dual nature of SAM. Hence, this molecule is rather considered to be a methylome modulator than a pure hypermethylating agent, based on the hypothesis suggested by Wang et al. and our recent study results as well [22,55].

Enrichment analysis revealed that KEGG signaling pathways tightly related to carcinogenesis (such as “PI3K-Akt” [56], “MAPK” [57], “Rap1” [58], “regulation of actin cytoskeleton” [59], “focal adhesion” [60], and “proteoglycans in cancer” [61]) were the most dominantly affected ones by DNA methylation changes. The former two of the above-mentioned processes have been reported to be influenced by the folic acid status [62,63]. Considering the distribution of DMSs in distinct genomic regions, we observed an enrichment mainly in heterochromatin (or low signal) sequences, similarly to other RRBS studies [64,65]. Besides these regions, active and weak promoter sequences were also represented dominantly.

The methylation status of promoter-associated CpG islands is of particular importance because it can modulate gene expression [21,42]; therefore, our study was complemented with genome-wide transcriptome analysis of FA-depleted and 10,000 ng/mL FA-treated samples. We observed that genes showing a decreased expression after FA treatment outnumbered the upregulated ones. In the SW480 cell line, not only the number of genes with altering expression (*p* *≤* 0.05 and FC ≥ |1.5|) but also the extent of alterations and the predicted functional associations between the encoded proteins were more prominent than in HT-29 cells. *YWHAZ* and the cytokine-expressing *TNF* were considered as the coding genes of hub proteins in the HT-29 and SW480 cell lines, respectively, both possessing significant (*p* *≤* 0.05) downregulation for FA supplementation. *YWHAZ* is a potential oncogene that can enhance proliferation, migration, and metastasis in many cancer types; therefore, its inhibition has strong clinical importance [66]. Moreover, ERK1/2 and PI3K/Akt/mTOR pathways mentioned in this study earlier have been reported to be the downstream targets of *YWHAZ* [66]. Not only the proliferation changes of HT-29 cells were supported by such gene expression alterations, but also the increased genomic stability. We examined the relevance of the top 10 up- and downregulated genes in DNA repair, and only *HES1* [67], *STAT3* [68], and *CCL2* [69] were reported to be involved in this process. The former two had an increased expression in HT-29 cells, while the latter was silenced in SW480 cells following the treatment, which could explain the different DNA repair abilities between the cell lines.

Finally, we combined the results of RRBS and microarray analyses to investigate whether FA actually affects gene expression by methylating or unmethylating the promoter regions. We observed that promoter methylation caused transcriptional silencing (*p* *≤* 0.05 and FC ≥ |1.5|) of four genes in HT-29 and five genes in SW480 cells following 10,000 ng/mL FA supplementation. On the other hand, promoter hypomethylation was accompanied by the upregulation (*p* *≤* 0.05 and FC ≥ |1.5|) of 10 and 8 genes in the HT-29 and SW480 cell lines, respectively. Due to the fact that the methylation status of the promoter region was not in concordance with the expression of numerous genes, we suppose that short-term FA treatment does not primarily exert its effect on gene regulation by altering promoter methylation. The involvement of other mechanisms, for example, histone methylation, can be implicated in this finding, as also hypothesized by Price et al. [70].

## 5. Conclusions

FA is a widely consumed vitamin known to be involved in an important epigenetic regulatory process, DNA methylation. However, our results suggest that in HT-29 and SW480 CRC cells, short-term FA supplementation can equally induce DNA hyper- and hypomethylation that mainly affects the pathways related to carcinogenesis. Promoter methylation changes could act on gene regulation, but we also suppose the involvement of other FA-associated effects behind the great number of differentially expressed genes. As opposed to SW480, FA treatment had a significant impact on the maintenance activities of HT-29 cells, including proliferation, viability, and DNA repair. Apart from the distinct molecular background between the two cell lines, expression alterations of genes contributing to the mentioned processes might explain this finding. Based on the results of this study, we can conclude that the short-term effect of FA on the observed cell lines goes far beyond the fundamental attributes connected to this vitamin.

## Data Availability

The data presented in this study were uploaded to the Gene Expression Omnibus (GEO) repository, reference number [GSE186084].

## Supplementary Materials

The following supporting information can be downloaded at: https://www.mdpi.com/article/10.3390/cancers14071820/s1, Figure S1: DAPI (blue) and anti-γ-H2AX (green) double-staining of HT-29 and SW480 cell lines; Figure S2: DNA integrity evaluation of HT-29 and SW480 cell lines with comet assay after exposing the samples to different folic acid (FA) concentrations (0, 100, 10,000 ng/mL); Figure S3: Principal component analysis (PCA) of Human Transcriptome Array 2.0 (HTA 2.0) gene expression data obtained from non-treated (0 ng/mL) and 10,000 ng/mL folic acid (FA)-supplemented HT-29 and SW480 cells; Table S1: The raw data of Figure 1, Figure 2 and Figure 3; Table S2: The list of the top 10 differentially expressed genes.
