# Supplementary material for: Folic Acid Treatment Directly Influences the Genetic and Epigenetic Regulation along with the Associated Cellular Maintenance Processes of HT-29 and SW480 Colorectal Cancer Cell Lines
Source: Cancers (Basel). 2022 Apr 3;14(7):1820. doi: 10.3390/cancers14071820 (PMC8997840; doi:10.3390/cancers14071820)
Supplement: Supplementary file 1 [file cancers-14-01820-s001.zip › Supplementary Figure S1.pdf]

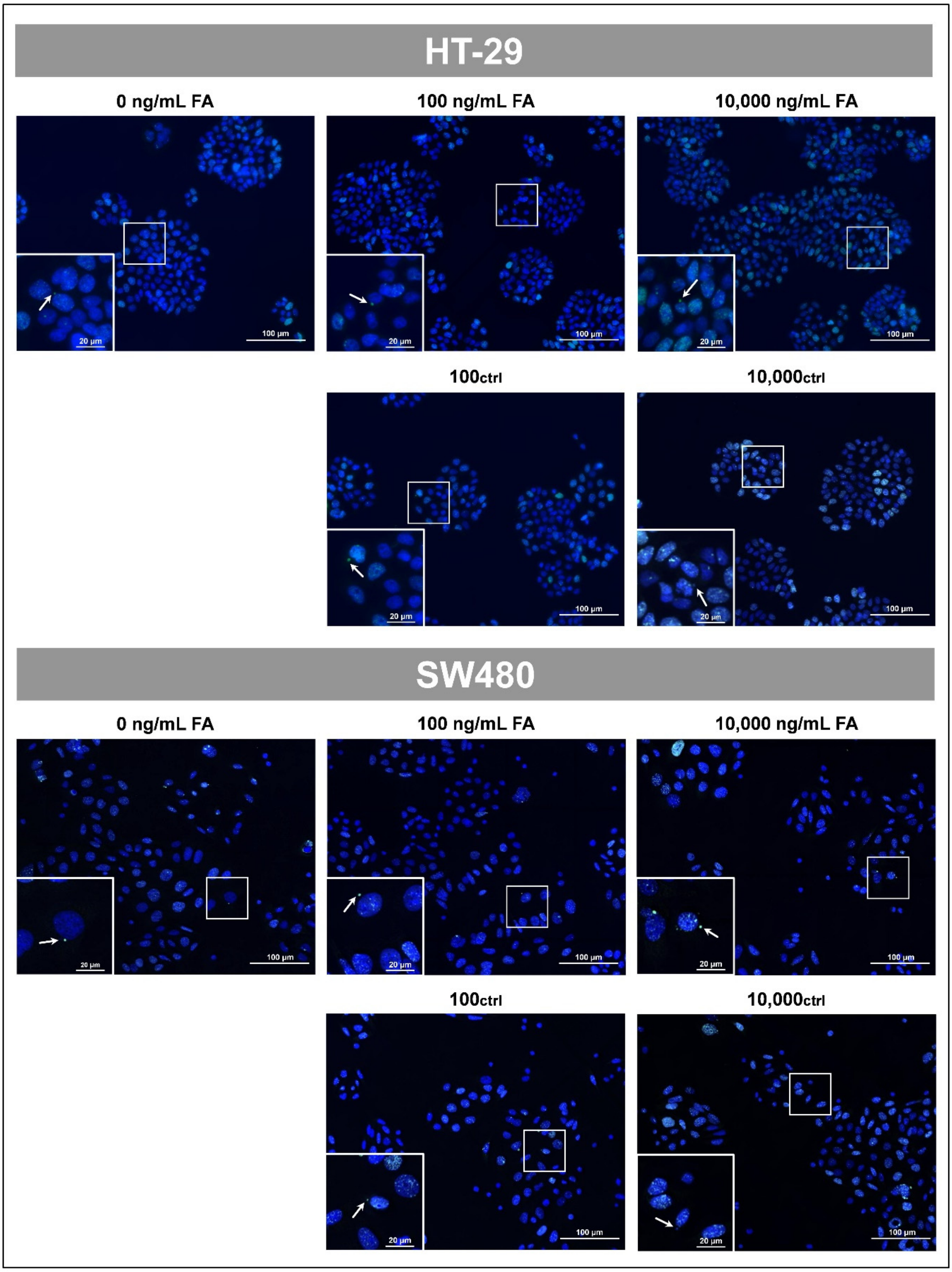

Figure S1. DAPI (blue) and anti-γ-H2AX (green) double-staining of HT-29 and SW480 cell lines. Genomic stability of non-treated (0 ng/mL) and folic acid (FA)-treated (100, 10,000 ng/mL) cells was detected with micronucleus scoring. FA was dissolved in 1 M NaOH before adding to the medium; therefore, we treated cells with 1M NaOH in the same amount used in the case of FA supplementation (100ctrl, 10,000ctrl) to detect its individual effect. Representative micronuclei are indicated with arrows.
