# Supplementary material for: Folic Acid Treatment Directly Influences the Genetic and Epigenetic Regulation along with the Associated Cellular Maintenance Processes of HT-29 and SW480 Colorectal Cancer Cell Lines
Source: Cancers (Basel). 2022 Apr 3;14(7):1820. doi: 10.3390/cancers14071820 (PMC8997840; doi:10.3390/cancers14071820)
Supplement: Supplementary file 1 [file cancers-14-01820-s001.zip › Supplementary Figure S2.pdf]

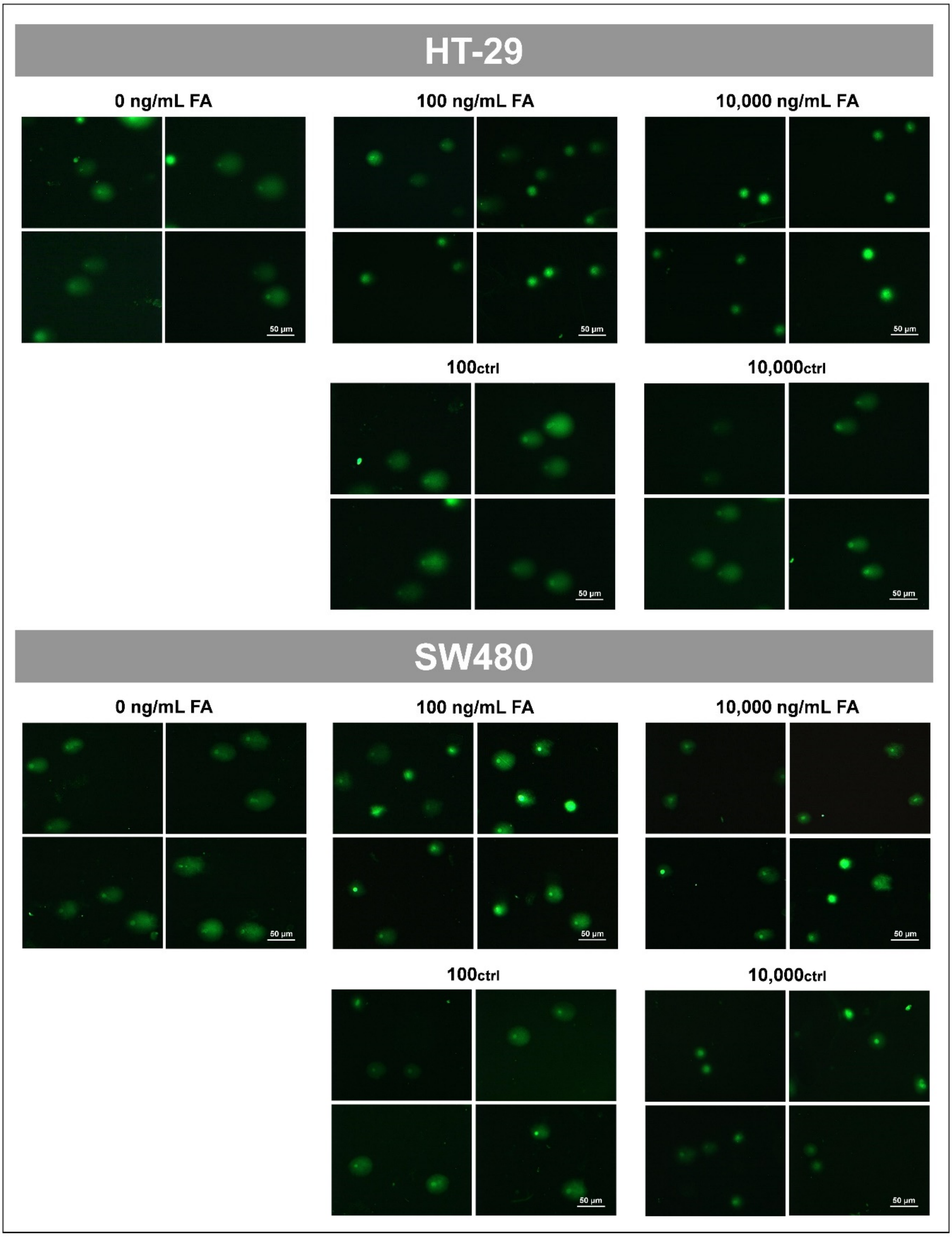

Figure S2. DNA integrity evaluation of HT-29 and SW480 cell lines with comet assay after exposing the samples to different folic acid (FA) concentrations (0, 100, 10,000 ng/mL). We dissolved FA in 1 M NaOH before it was added to the medium; therefore, cells were treated with 1M NaOH in the same amount used in the case of FA supplementation (100ctrl, 10,000ctrl) to detect its individual effect.
