# Supplementary material for: Folic Acid Treatment Directly Influences the Genetic and Epigenetic Regulation along with the Associated Cellular Maintenance Processes of HT-29 and SW480 Colorectal Cancer Cell Lines
Source: Cancers (Basel). 2022 Apr 3;14(7):1820. doi: 10.3390/cancers14071820 (PMC8997840; doi:10.3390/cancers14071820)
Supplement: Supplementary file 1 [file cancers-14-01820-s001.zip › Supplementary Figure S3.pdf]

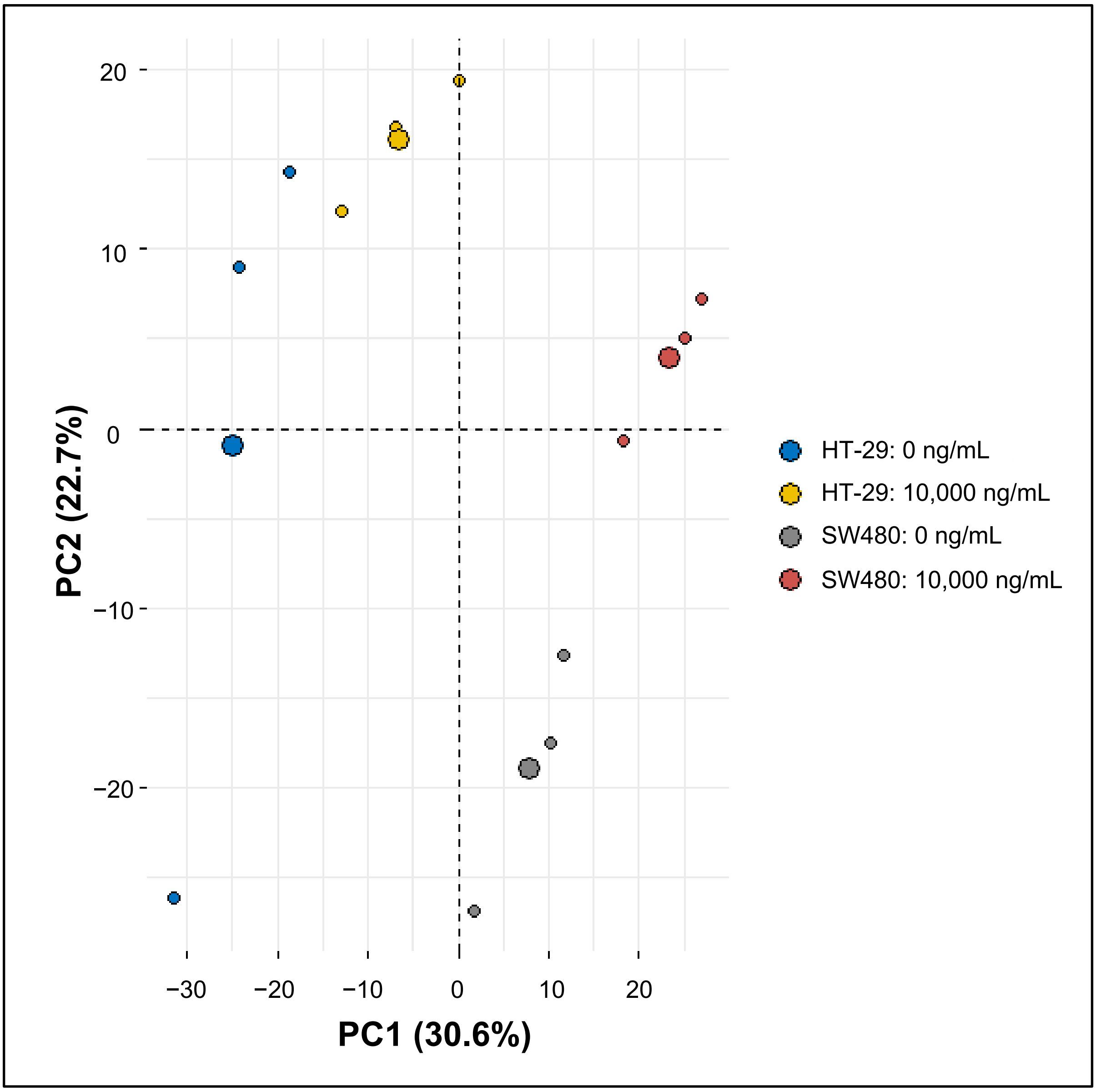


**Figure S3.** Principal component analysis (PCA) of Human Transcriptome Array 2.0 (HTA 2.0) gene expression data obtained from non-treated (0 ng/mL) and 10,000 ng/mL folic acid (FA)-supplemented HT-29 and SW480 cells. Only genes showing significant (*p*≤0.05) expression alterations with a ≥ |1.5| fold change (FC) were evaluated. Small data points represent individual microarray samples, while large data points refer to the average of each sample group. PC: principal component.
